# Molecular diversity of clustered protocadherin-α required for sensory integration and short-term memory in mice

**DOI:** 10.1038/s41598-018-28034-4

**Published:** 2018-06-25

**Authors:** Tatsuya Yamagishi, Kohei Yoshitake, Daiki Kamatani, Kenji Watanabe, Hiroaki Tsukano, Ryuichi Hishida, Kuniyuki Takahashi, Sugata Takahashi, Arata Horii, Takeshi Yagi, Katsuei Shibuki

**Affiliations:** 1 Department of Neurophysiology, Brain Research Institute, Niigata University, 1-757 Asahi-machi, Chuo-ku, Niigata 951-8585 Japan; 2 Department of Otolaryngology Head and Neck Surgery, Graduate School of Medical and Dental Sciences, Niigata University, 1-757 Asahi-machi, Chuo-ku, Niigata 951-8510 Japan; 3 KOKORO-Biology Group, Graduate School of Frontier Biosciences, Osaka University, 1-3 Yamadaoka, Suita, 565-0871 Osaka Japan; 4 The Japan Agency for Medical Research and Development, 1-3 Yamadaoka, Suita, 565-0871 Osaka Japan

## Abstract

Clustered protocadherins (Pcdhs) are neuronal cell adhesion molecules characterized by homophilic adhesion between the tetramers of 58 distinct isoforms in mice. The diversity of Pcdhs and resulting highly-specific neuronal adhesion may be required for the formation of neural circuits for executing higher brain functions. However, this hypothesis remains to be tested, because knockout of *Pcdh* genes produces abnormalities that may interfere with higher brain functions indirectly. In *Pcdh-α1*,*12* mice, only α1, α12 and two constitutive isoforms are expressed out of 14 isoforms. The appearance and behavior of *Pcdh-α1*,*12* mice are similar to those of wild-type mice, and most abnormalities reported in *Pcdh-α* knockout mice are not present in *Pcdh-α1*,*12* mice. We examined *Pcdh-α1*,*12* mice in detail, and found that cortical depression induced by sensory mismatches between vision and whisker sensation in the visual cortex was impaired. Since Pcdh-α is densely distributed over the cerebral cortex, various types of higher function are likely impaired in *Pcdh-α1*,*12* mice. As expected, visual short-term memory of space/shape was impaired in behavioral experiments using space/shape cues. Furthermore, behavioral learning based on audio-visual associative memory was also impaired. These results indicate that the molecular diversity of Pcdh-α plays essential roles for sensory integration and short-term memory.

## Introduction

Mouse models are powerful tools for investigating mental disorders^1^, some of which are likely attributed to abnormalities in complex neural circuits required for executing higher brain functions. Clustered protocadherins (Pcdhs) are neuronal cell adhesion molecules with 58 distinct isoforms coded by *Pcdh-α*, *-β* and *-γ* gene clusters in mice^2,3^. A few isoforms in each gene cluster are randomly selected by stochastic expression at the individual neuron level, and the resulting diversity in Pcdhs is expected to play an important role in the formation of complex neural circuits^4–6^, which possibly execute higher brain functions. However, this possibility is difficult to test. Mice lacking *Pcdh-γ* are born alive, though they die within 12 h after birth^7^. The loss of *Pcdh-α* or *-β* results in several non-lethal abnormalities^8–13^, which may interfere with higher brain functions indirectly.

The roles of Pcdh diversity in higher brain functions may be elucidated by investigating genetically-manipulated mice, in which the diversity of Pcdhs is reduced yet no apparent abnormality is found. *Pcdh-α1*,*12* mice express only α1 and α12 out of the variable α1–12 isoforms, along with an intact expression of constitutive Pcdh-α isoforms^14–16^. In *Pcdh-α1*,*12* mice, α1 and α12 increase in a compensatory manner; thus, the total amount of Pcdh-α remains constant^16^. The appearance and behavior of *Pcdh-α1*,*12* mice are similar to those of wild-type mice, and most abnormalities found in *Pcdh-α* knockout mice are not present in *Pcdh-α1*,*12* mice, except for a particular type of cortical plasticity dependent on multimodal sensory integration^12^. We further investigated *Pcdh-α1*,*12* mice in detail and found that two types of higher functions based on multimodal sensory integration and two types of visual short-term memory were impaired. These findings clearly indicate that diversity of Pcdh-α is required for executing higher cognitive functions including short-term memory and sensory integration, possibly because highly specific homophilic interactions between the tetramers of Pcdhs are required for specificity in Pcdh-dependent circuit formation together with growth of a properly complex dendrite arbor^17^.

## Results

### Visual-whisker integration

**Figure 1:**
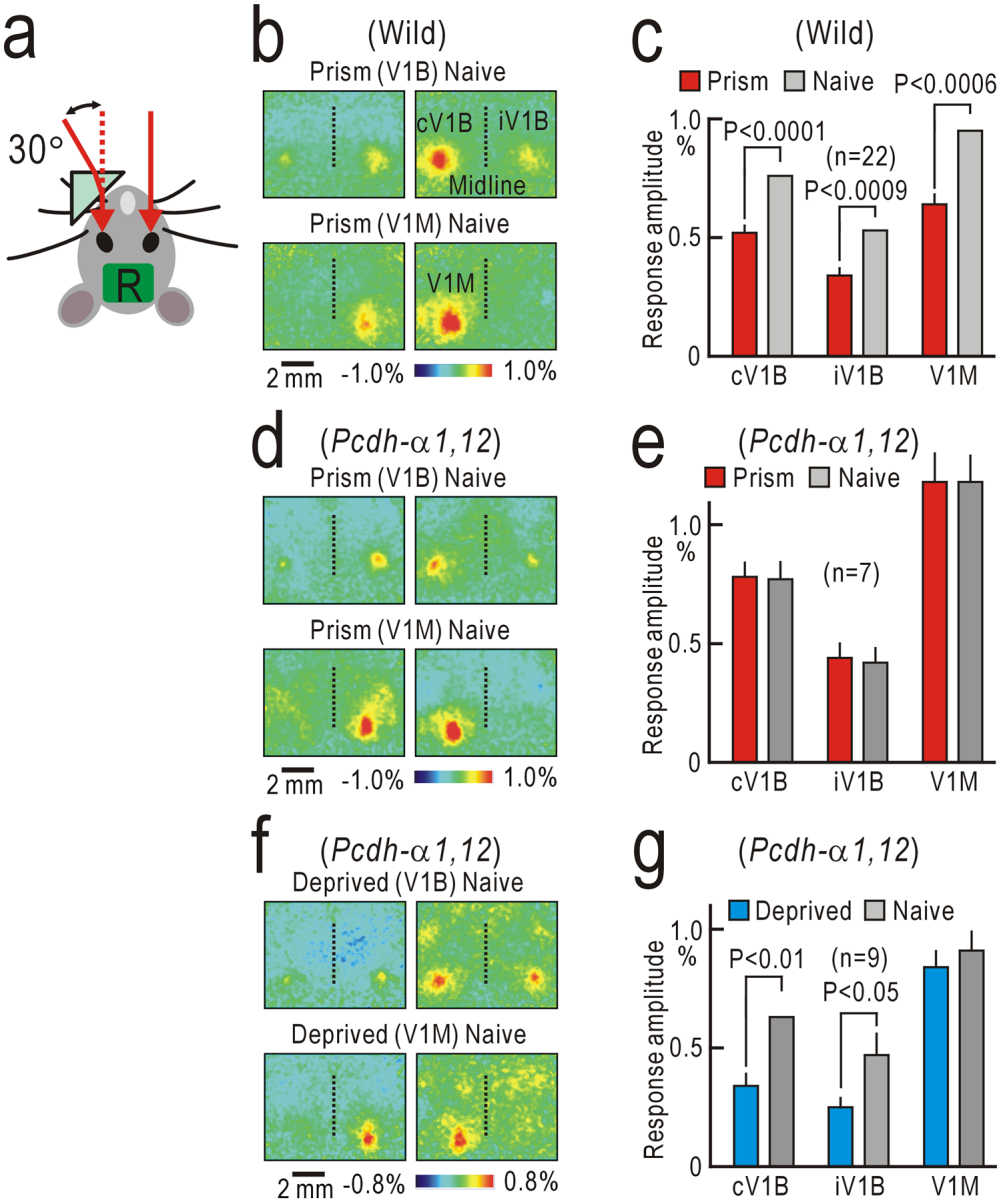
Mismatch-induced cortical depression in the V1. (**a)** Monocular prism goggle for inducing cortical depression that requires multimodal sensory mismatch between vision and whisker sensations. R represents the imaged areas, including the bilateral V1. (**b)** Mismatch-induced depression in a wild-type mouse. Images were obtained after removing the prism goggle. The upper two panels show bilateral cortical responses in the binocular regions of the V1 (V1B). Responses were elicited by visual stimulation to the left eye that had worn the monocular prism goggle (left) and the right naïve eye (right). The lower two panels are the responses in the monocular region of the V1 (V1M). (**c)** Histograms of response amplitudes of the contralateral responses in the V1B (cV1B), and ipsilateral responses in the V1B (iV1B) and V1M. (**d**,**e**) Absence of mismatch-induced depression in a *Pcdh-α1*,*12* mouse. Data in (**b**) and (**c**) were reproduced from our previous study^12^. (**f**,**g**) Ocular dominance plasticity after monocular deprivation in a *Pcdh-α1*,*12* mouse. Significant depression was observed in the cV1B and iV1B, but not the V1M.

The appearance and behavior of *Pcdh-α1*,*12* mice were similar to those of wild-type mice (Video 1). We investigated cortical depression in the primary visual cortex (V1) induced by spatial mismatches between vision and whisker sensation, since this depression is impaired in *Pcdh-α* knockout mice^12^. To produce multimodal sensory mismatches, a small monocular prism goggle was attached in front of the left eye (Fig. 1a). After the goggle was removed, visual responses were recorded. When light stimuli were placed in front of the left eye, cortical responses were observed in the contralateral binocular region of the V1 (cV1B) and the ipsilateral binocular region of the V1 (iV1B) (Fig. 1b, left upper panel). The cortical responses in the monocular region of the V1 (V1M) were elicited by light stimuli placed on the left side of the mouse (Fig. 1b, left lower panel). These responses elicited via the left prism eye were weaker than the corresponding responses elicited via the right naive eye (Fig. 1b, right panels). The amplitudes of the cortical responses elicited via the left prism eye were significantly depressed compared to those elicited via the right naive eye in the cV1B, iV1B, and V1M (Fig. 1c). These results indicate that prism-induced depression was present in the V1M as well as in the V1B^12^. We performed similar experiments in *Pcdh-α1*,*12* mice. However, no clear depression was observed in the V1M and V1B (Fig. 1d,e). In contrast, ocular dominance plasticity was observed after monocular deprivation in *Pcdh-α1*,*12* (Fig. 1f,g), as in wild-type mice^18^. Single neuronal activities in the V1 were recorded using two-photon Ca^2+^ imaging. However, we found no apparent difference in orientation/direction selectivity of V1 neurons between wild-type and *Pcdh-α1*,*12* mice (Fig. S1).

### Visual short-term memory of spatial information

**Figure 2:**
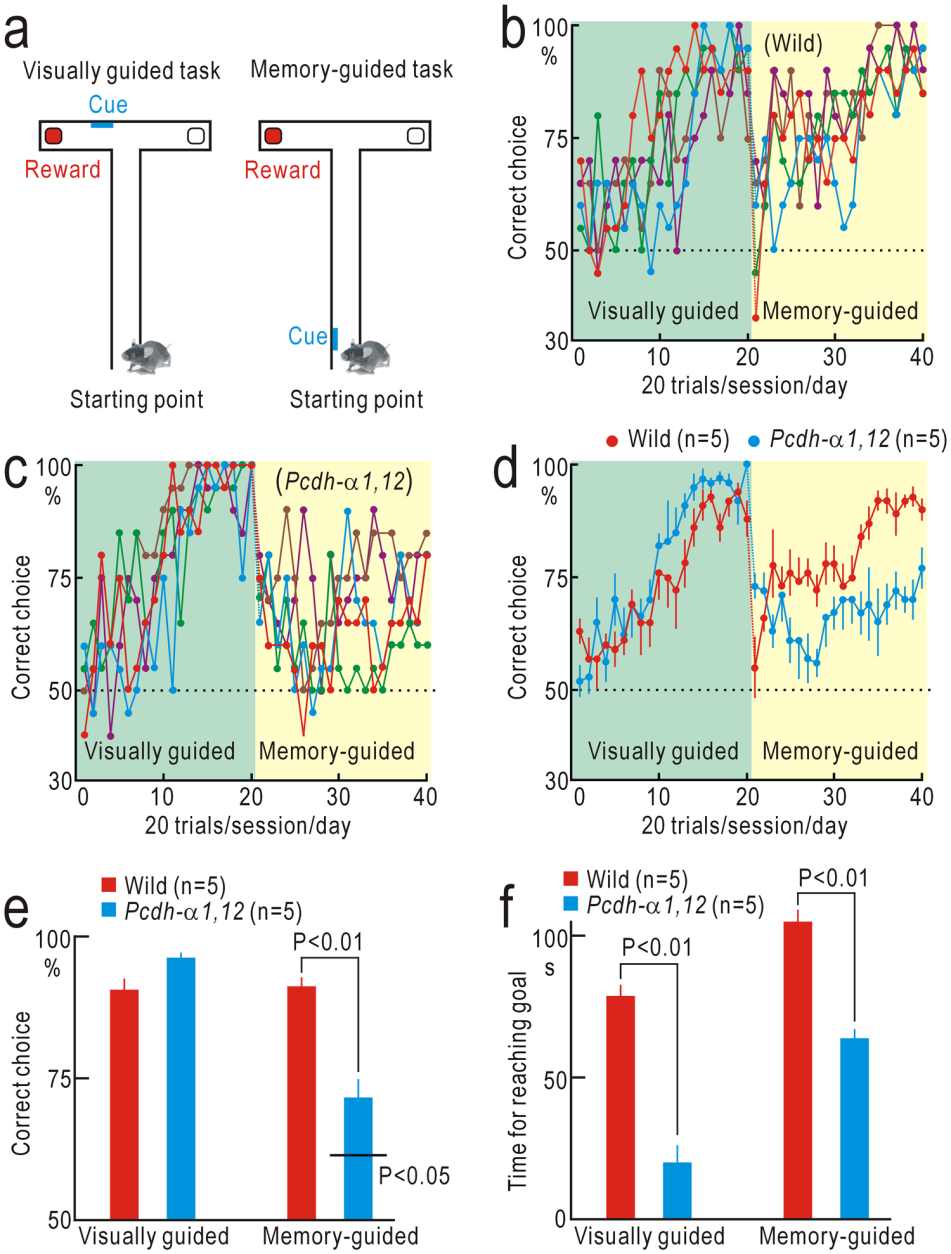
Short-term memory for spatial information. (**a)** T-maze test. (**b)** Performance of five wild-type mice. (**c)**, Performance of five *Pcdh-α1*,*12* mice. (**d)** The mean and SEM. of data shown in (**b**) and (**c**). (**e**) Averaged amplitudes of performance in the last five sessions of the visually guided and memory-guided tasks. The performance of *Pcdh-α1*,*12* mice in the memory-guided task was significantly worse than that of wild-type mice (P < 0.01), though better than chance level (P < 0.05). (**f**) Time required for reaching the correct goals. Significantly less time was spent by *Pcdh-α1*,*12* mice in the visually- and memory-guided tasks.

Spatial information obtained from vision and whisker sensation is thought to be integrated in the posterior parietal cortex (PPC) for detecting multimodal sensory mismatch^12^. The PPC is reported to play additional roles in terms of visual short-term memory for spatial information^19^. Therefore, visual short-term memory may also be impaired in *Pcdh-α1*,*12* mice. We tested this possibility using a T-maze test (Fig. 2a). First, mice were trained with a visually guided task as a control. Wild-type mice learned to choose the correct branches marked with a visual cue to receive a reward. The task performance reached a plateau level within 20 days/sessions (Fig. 2b). The performance of *Pcdh-α1*,*12* mice in the visually guided task was similar to that of wild-type mice (Fig. 2c,e). After the performance in the visually guided task reached a plateau level, we moved the position of the visual cue to the left or right wall of the T-maze near the starting point. Under this condition, short-term memory of the right or left cue position was required for the successful choice of rewarded branches (Fig. 2a). The performance of wild-type mice in this memory-guided task was suppressed immediately after task switching down to chance level, and then gradually recovered up to the performance level before task switching (Fig. 2b,d). The performance of *Pcdh-α1*,*12* mice was also suppressed immediately after task switching; however, the suppression was mild compared to that in wild-type mice. The performance level did not recover afterwards (Fig. 2c,d), and the final performance of *Pcdh-α1*,*12* mice (n = 5) was significantly worse than that of wild-type mice (n = 5, P < 0.01, Fig. 2e). Interestingly, *Pcdh-α1*,*12* mice reached the goal area faster than wild-type mice in the visually guided task and the memory-guided task (Fig. 2f), suggesting that *Pcdh-α1*,*12* mice spent less time in the decision-making.

### Visual short-term memory of shape information

**Figure 3:**
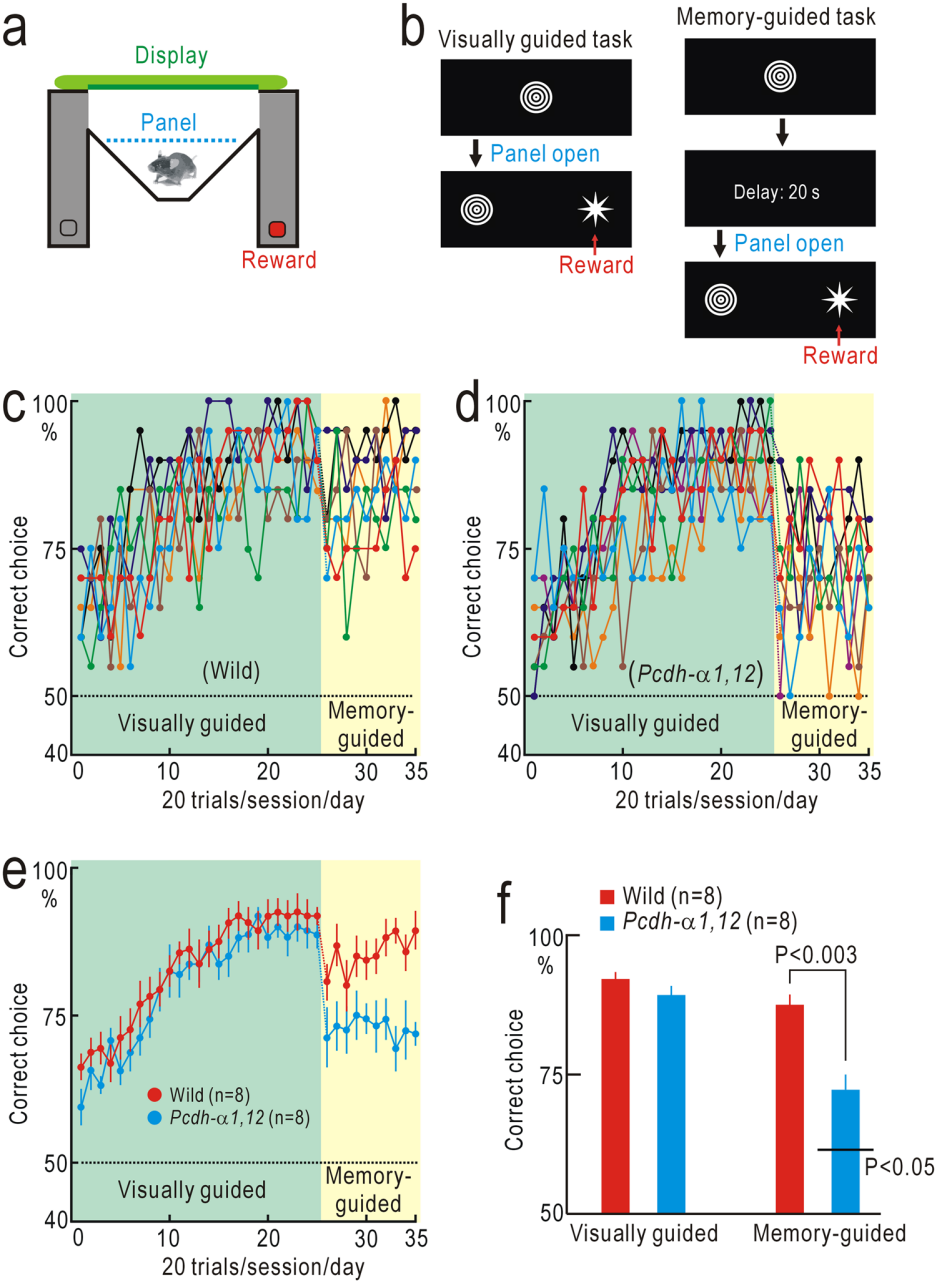
Short-term memory for shape information. (**a**) M-shaped maze. (**b**) Visually guided and memory-guided tasks. A nonmatching-to-sample test with or without a delay of 20 s was used. (**c**) Performance of eight wild-type mice. (**d**) Performance of eight *Pcdh-α1*,*12* mice. (**e**) The mean and SEM of data are shown in (**c**) and (**g**). (**f**) Averaged amplitudes of performance in the last five sessions (100 trials) of the visually guided and memory-guided tasks. The performance of *Pcdh-α1*,*12* mice in the memory-guided task was significantly worse than that of wild-type mice.

In primates, higher visual areas are divided into a dorsal pathway specialized in motion/spatial information processing and a ventral pathway specialized in pattern/shape information processing^20^. Mice have visual areas surrounding the V1, and these higher areas are considered to be homologous to those of primates^21–23^. The wide distribution of Pcdh-α throughout the cerebral cortex^16^ suggests that short-term memory of pattern/shape information can also be affected in *Pcdh-α1*,*12* mice. To test this possibility, we constructed an M-shaped maze to investigate short-term memory of shape information (Fig. 3a,b). First, mice were trained with a visually guided task as a control. Wild-type mice learned to choose the correct branches marked with the choice shapes that differed from those in sample images (non-matching-to-sample test, Fig. 3b). The performance in this test reached plateau level after 20 days/sessions of training (Fig. 3c,e). Next, we introduced a delay period of 20 s between the presentation of the sample and choice images (Fig. 3b). The performance of wild-type mice in this memory-guided task was suppressed immediately after task switching, and gradually recovered up to the performance level before task switching (Fig. 3c,e). Similarly, wild-type mice could perform a delayed matching-to-sample test, and finally they could discriminate even between a pair of alphabets (Fig. S2). The performance of *Pcdh-α1*,*12* mice in the visually guided task was similar to that of wild-type mice (Fig. 3d,e), and no significant difference was observed in the performance level between wild-type and *Pcdh-α1*,*12* mice (Fig. 3f). The performance of *Pcdh-α1*,*12* mice was also suppressed immediately after task switching, although no clear performance recovery was observed afterward (Fig. 3d,e). The final performance level of *Pcdh-α1*,*12* mice (n = 8) with a delay of 20 s was better than chance level (P < 0.05) in the last five sessions (100 trials), although significantly worse than that of wild-type mice (n = 8, P < 0.003, Fig. 3f). Taken together with the results shown in Fig. 2, it is indicated that visual short-term memory is impaired in *Pcdh-α1*,*12* mice.

### Audio-visual integration

**Figure 4:**
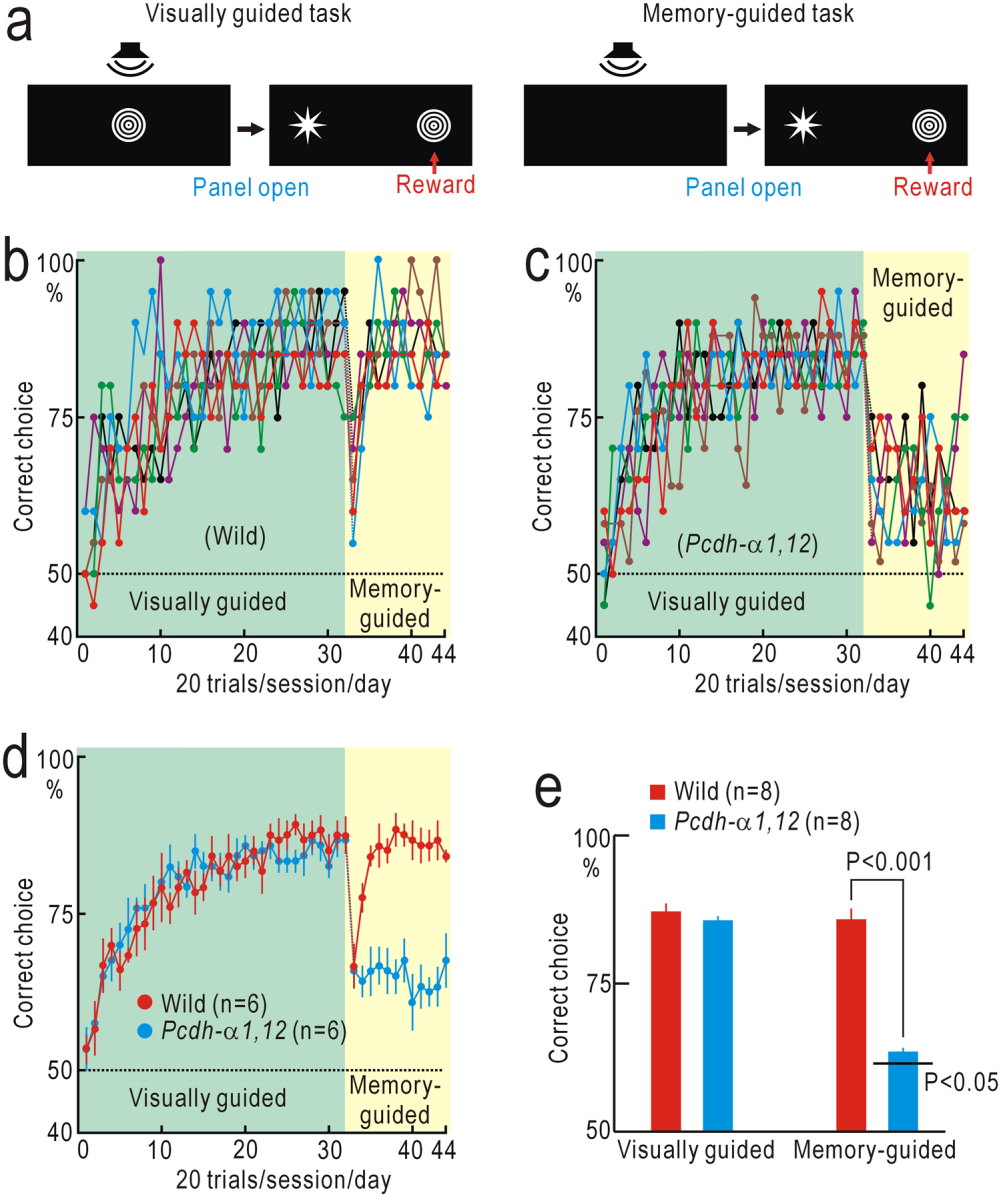
Audio-visual associative learning. (**a**) Tasks in the experiments. The visually guided task was a matching-to-sample test, in which presentation of a visual sample was coupled with the presentation of a specific sound cue. In the memory-guided task, mice were required to select visual samples based on sound cues only. (**b**) Performance of six wild-type mice. (**c**) Performance of six *Pcdh-α1*,*12* mice. (**d**) The mean and SEM of the data shown in (**b**) and (**c**). (**e)** Averaged amplitudes of performance in the last five sessions in the visually guided and memory-guided tasks. The performance of *Pcdh-α1*,*12* mice in the memory-guided task was significantly worse than that of wild-type mice.

From the results shown in Fig. 1, we speculated that various types of multimodal sensory integration may be impaired in *Pcdh-α1*,*12* mice. Fortunately, the M-shaped maze was appropriate to investigate associative memory that depends on Audio-visual integration. First, mice were trained with a visually guided task using a sample-to-match test with no delay. During this phase, the presentation of each sample shape was combined with the presentation of a specific sound cue in a one-to-one manner (Fig. 4a, Video 2). The performance of wild-type mice and *Pcdh-α1*,*12* mice reached a plateau level within 20–30 days/sessions in this test (Fig. 4b–d), and no significant difference was found in the performance level between the two groups of mice (Fig. 4e). In the next stage, mice had to choose the correct shapes based on associative memory between shapes and the corresponding sound cues (Video 3). The performance of wild-type mice was transiently suppressed immediately after task switching, and then gradually recovered up to the performance level before task switching (Fig. 4b,d). Similarly the performance of *Pcdh-α1*,*12* mice was suppressed, yet almost no recovery was observed afterward (Fig. 4c,d). The final performance level of *Pcdh-α1*,*12* mice (n = 8) in the associative memory-guided task was better than chance level (P < 0.05) in the last five sessions (100 trials), although significantly worse than that of wild-type mice (n = 8, P < 0.001, Fig. 4e).

## Discussion

In the present study, we found four types of abnormal phenotypes related to higher cognitive functions in *Pcdh-α1*,*12* mice. In behavioral experiments analyzing visual short-term memory, we investigated visually guided tasks first. The performance of *Pcdh-α1*,*12* mice was similar to that of wild-type mice. Moreover, ocular dominance plasticity, orientation selectivity, and direction selectivity of V1 neurons were normal in *Pcdh-α1*,*12* mice. These results indicate that visual functions and learning abilities are not impaired in *Pcdh-α1*,*12* mice. Second, we tested the memory-guided tasks, in which the performance of *Pcdh-α1*,*12* mice was inferior to that of wild-type mice. These results indicate that visual short-term memory is impaired in *Pcdh-α1*,*12* mice. The suppression of mismatch-induced cortical plasticity and the poor behavioral performance based on Audio-visual associative memory suggest that multimodal sensory integration is also impaired in *Pcdh-α1*,*12* mice. Considering the extensive distribution of Pcdh-α in most cortical neurons^16^, a wide variety of higher cognitive functions can be related to Pcdh-α. *Pcdh-α1*,*12* mice seemed to be more determined in decision-making compared to wild-type mice, since *Pcdh-α1*,*12* mice spent less time to reach the correct goals in the T-maze test. In the memory-guided tasks, *Pcdh-α1*,*12* mice quickly reached the final performance level, which was low yet significantly better than chance level. In contrast, the final performance level was achieved only 5–15 days/sessions after task switching in wild-type mice. This difference might be explained by impairment of cognitive flexibility in *Pcdh-α1*,*12* mice. Cognitive flexibility is usually required for reversal learning^24,25^ and is controlled by serotonergic neurons^26^. The present behavioral tests did not include reversal learning. In *Pcdh-α* knockout mice, abnormal distribution of serotonergic fibers is found^10^, and this phenotype is attributed to loss of αc2, a constitutively-expressed Pcdh-α isoform in serotonergic neurons^27^. Therefore, it is possible that the abnormalities in *Pcdh-α* knockout mice may be derived from a reduced diversity of Pcdh-α, or a loss of constitutive isoforms such as αc1 and αc2. Since constitutive Pcdh-α isoforms are intact in *Pcdh-α1*,*12* mice^16^, it is suggested that the four phenotypes were derived from the reduced diversity of Pcdh-α.

Short-term memory is maintained by persistent neural firing for more than a few seconds after the offset of stimuli^28–31^. Various mechanisms underlying persistent firing have been proposed including roles for intrinsic neuronal properties^32–35^, short-term synaptic potentiation^36–38^, and specialized neural circuits^39,40^. Possibly, short-term memory may require a combination of these mechanism^30,31^. The present results can provide insight into the neural mechanisms of short-term memory by highlighting the possible roles of Pcdhs. The molecular diversity of Pcdhs plays a role in self-avoidance in dendritic synapse formation^4,41^. In the synapses between different neurons, the molecular diversity of Pcdhs is considered necessary for specific circuit formation^5,6^. This suggests that the specificity of Pcdh-dependent synaptic formation deteriorates as the molecular diversity of Pcdh-α decreases, and that the formation of specialized neural circuits required for short-term memory may be impaired in *Pcdh-α1*,*12* mice. In addition, synaptic mechanisms for short-term memory require sparse readout circuits that convert synaptic potentiation to neural activities with minimal dissipation of information^36–38^, and such sparseness of readout circuits may be deteriorated in *Pcdh-α1*,*12* mice. Assuming that two neuronal layers are sparsely connected in a one-to-one fashion, localized input to the first layer produces short-term potentiation (STP) in the stimulated synapses (Fig. S4). The information stored as STP can be converted to short-term memory by adding diffuse input to the first layer. However, the content in short-term memory is obscured in *Pcdh-α1*,*12* mice. Taken together, the present results suggest that accurate and sparse synaptic formation controlled by the molecular diversity of Pcdh-α plays an important role in the neural mechanisms underlying short-term memory.

The neural mechanisms underlying the integration of information are largely unknown. The impairment of short-term memory and sensory integration in *Pcdh-α1*,*12* mice suggests that a common neural mechanism may exist for both functions. NMDA receptors can induce both long-term potentiation and STP^42,43^ and play an essential role in short-term memory^32^, as well as in associative learning^44^. Therefore, NMDA receptor-dependent STP can serve as a common mechanism for short-term memory and associative learning. Hebb proposed that formation of a functionally connected dynamic cell assembly of neurons representing each aspect of information may explain how information is integrated as a whole^45^, while it is unknown how such dynamic cell assemblies are stably present in the brain. Neural firing representing short-term memory is not necessarily continuous^38^, and sequential activities of multiple neurons have been observed during the maintenance of short-term memory^19^, suggesting a transfer of contents between short-term memory circuits. When short-term memory circuits storing each aspect of information are connected with content transfers, a stable form of dynamic cell assembly can be formed. NMDA receptor-dependent potentiation works as spike-timing dependent plasticity^46^, in which synchronized bursting activities underlying short-term memory^47^ can be integrated. Therefore, assuming the involvement of NMDA receptors, short-term memory circuits may also play a role in the integration of information. Although these mechanisms remain to be confirmed, the present results indicate the importance of Pcdh-dependent neural circuit formation for sensory integration. A wide variety of higher cognitive functions can be related to Pcdh-α, as has been reported in humans^48,49^. Possibly, even higher mental functions such as consciousness could also be dependent on the diversity of Pcdhs, since short-term memory and integration of information are essential components of consciousness^50^.

## Materials and Methods

### Ethical approval and animals

The ethics committee of Niigata University approved the experimental protocols used in this study (approval numbers: 372-7 and SA00143). All experiments were performed in accordance with approved guidelines and regulations. C57BL/6 mice and *Pcdh-α1*,*12* mice^16^ of both sexes were used.

### Endogenous fluorescence imaging of cortical activities

Activity-dependent changes in endogenous fluorescence derived from mitochondrial flavoproteins^51^ were used for imaging cortical activities in the V1, because this imaging technique requires no exogenous fluorophore and has been proven appropriate for examining cortical plasticity in mice^12,18^. Mice were anesthetized with urethane (1.6 g/kg, i.p.). Throughout recordings, rectal temperature was maintained at 38 °C using a silicon rubber heater. Surgical procedures were conducted under sterile conditions. After subcutaneous injection of bupivacaine (AstraZeneca, Osaka, Japan), the disinfected skin was incised, and the skull covering the imaged area was exposed. The surface of the intact skull was covered with a mixture of liquid paraffin and Vaseline to prevent drying and to keep the skull transparent. The head was fixed using a stereotaxic frame (SG-4, Narishige, Tokyo, Japan). Surgical procedures usually completed within 20 min. Imaging was conducted approximately 60 min after administration of urethane. An additional dose of urethane (0.2 g/kg, S.C.) was administered when necessary. At the end of the imaging experiments, the mice were euthanized with an overdose of pentobarbital (i.p.).

Cortical images (128 × 168 pixels after binning) were recorded at nine frames/s using a cooled CCD camera system (Aquacosmos system with an ORCA-ER camera, Hamamatsu Photonics, Hamamatsu, Japan). The camera was attached to a binocular epifluorescence microscope with a 75 W xenon light source (MZ FL III, Leica Microsystems, Wetzlar, Germany). Fluorescence images (λ: 500–550 nm) excited by blue light (λ: 450–490 nm) were acquired while the mice were administered visual stimuli in trials repeated at 20 s intervals. Images were averaged over 24–40 trials. Spatial moving averaging in 5 × 5 pixel areas was also used. Images were normalized, pixel by pixel, with respect to a reference image, which was obtained by averaging five images obtained immediately before stimulation. The normalized images are shown with a pseudocolour scale representing relative fluorescence changes (ΔF/F0). The response amplitude at 0.6–1.0 s after stimulus onset was evaluated as values of ΔF/F0 in a square window of 10 × 10 pixels. The location of the window was adjusted so that the response amplitude in ΔF/F0 was maximal.

### Sensory stimulation

During the recording of V1 responses, the stimulated eyes were opened until the entire pupil was exposed. The corneas were repeatedly covered with saline throughout the experiments to prevent drying. As a visual stimulus for evaluating V1 responses, we used a red LED (λ, 613 nm; diameter, 3 mm; TLSH160, Toshiba, Tokyo, Japan) placed 30 cm away from the mouse in the horizontal plane. The LED was turned on for 1 s in each trial, and only on-responses were examined. One of the two eyes was covered to enable stimulation only of the uncovered eye. LED stimuli were presented directly in front of the subject (0°) to elicit responses in the cV1B and iV1B, and on the left/right side (90°) to elicit responses in the V1M. For evaluating the properties of V1 neurons using two photon imaging, moving grating patterns were produced with a visual stimulus generator (ViSaGe, Cambridge Research System, Cheshire, UK), and presented on an 8-inch display (LCD-8000, Century, Tokyo, Japan) placed 11.5 cm away from the mice at an angle of 60° to the left. The properties of the grating patterns were as followings: 0.05 cycle/°, square wave red/black contrast, speed at 20°/s, eight directions from −45° to 270° in 45° steps, duration of 2 s. To avoid perturbation of fluorescence measurements using blue and green lights, the surface of the monitor was covered with a filter passing red light with λ > 600 nm (Sharp Cut Filter, Kenko, Tokyo Japan). For investigating Audio-visual integration, an auditory stimulus mimicking the sound of a cat’s meow was obtained from a free online source (http://taira-komori.jpn.org/index.html). We also used a sound mimicking a mouse squeak (http://on-jin.com/sound/ani.php). The peak intensity of these sound stimuli was adjusted to approximately 70 dB sound pressure level.

### Induction of cortical plasticity in V1

The surgical procedures required for producing cortical plasticity in V1 were conducted, as described previously^12,18^. Wild-type mice and *Pcdh-α1*,*12* mice at 4 weeks old were anesthetized with pentobarbital (60 mg/kg, i.p.). Fradiomycin and ampicillin were used to protect against infection. A clear acryl prism goggle weighing 0.6 g was attached to the skull with acrylic dental resin (Super Bond, Sun Medical, Shiga, Japan), so that the prism was located in front of the left eye. When ocular dominance plasticity was tested, the skin around the left eye was disinfected with 70% alcohol. Eyelids were sutured with a fine surgical nylon thread (diameter: 0.23 mm, Mani Inc., Tochigi, Japan). During monocular deprivation, mice were examined daily to ensure that the eyes remained closed and uninfected. An ophthalmic solution containing levofloxacin (5 mg/ml, Santen Pharmaceutical, Osaka, Japan) was applied to the sutured eye every day. Cortical plasticity was assessed 4–7 days after the surgical manipulation.

### Behavioral experiments

For testing short-term memory of visual spatial information, a T-maze was constructed with pieces of black plastic plate (Fig. 2a). The width, length, and height of the maze were 30 cm, 100 cm, and 10 cm, respectively. The width of the passage was 5 cm. As a visual cue, a white plastic plate (5 cm width × 10 cm height) was attached at the branching point in the visually guided task or at 10 cm from the starting point of the maze in the memory-guided task. The training and tests began when mice were 5–6 weeks old, and continued for up to 3 months. As a reward, a 50 μl drop of 5% glucose solution, on a plastic weighing dish placed at the end of the correct branch, was provided for each correct trial. In case of an error trial, mice repeated the trial until they selected the correct branch. When mice did not select any branch within 4 min after they were placed at the starting point of the maze, the trial was aborted. Each mouse underwent 20 trials per day. Therefore, each mouse consumed 1 ml of 5% glucose solution in each session. To maintain motivation for the reward, mice were reared under a scheme of intermittent water deprivation. In this scheme, the first session of the T-maze test was started 2 days after the initiation of water deprivation. Mice were trained for a maximum of 5 successive days before water deprivation was stopped. During the training in this scheme, dry food pellets were freely available. The body weight of the mice was maintained at approximately 85% of the weight before water deprivation, and normal body growth was observed throughout the intermittent water deprivation period (Fig. S2e).

For testing short-term memory of visual shape information, an M-shaped maze was constructed with pieces of black plastic plate (Fig. 3a). The width, length, and height of the maze were 32 cm, 21 cm and 10 cm, respectively. The upper boundary of the maze was closed with a tablet PC with a 10.1-inch display (PadTF700T, ASUS, Taipei, Taiwan) for visual stimulation. The central area of the maze was divided into two parts with a transparent plastic panel (Fig. 3a). The part distant from the display was used as a waiting area, where mice saw the display of the tablet PC. The transparent panel was removed by experimenters at the appropriate time, and mice selected one of the two branches connected to the part near the display. The two branches were maintained dark with a black plastic cover to facilitate choice by mice. In error trials, mice repeated the trial until they selected the correct branches. When mice did not select any branch within 20 s after the transparent panel was removed, the trial was aborted. Each mouse was tested in 20 trials per day. Therefore, each mouse consumed 1 ml of 5% glucose solution in each session. An intermittent water deprivation scheme was also used in this experiment. Visual stimuli were prepared using PowerPoint (Microsoft) and stored as videos for use on the tablet PC. In the visually guided task, a sample image of a circle or star (approximately 20° in diameter) was presented in the middle of the display for 10 s. The brightness of the sample images was modulated at 1 Hz using the animation function in PowerPoint. Immediately after presentation of the sample image, choice images of either a circle or a star were shown for 20 s at the entrance of each branch. In a matching-to-sample test, the branch marked with the same shape as the sample image was the correct choice. In a nonmatching-to-sample test, the branch marked with a different shape from the sample image was the correct choice. Mice were successfully trained to perform both tests, while the percentage of the correct choices was slightly higher in the nonmatching-to-sample test. Selection of the sample and choice images was determined randomly.

The M-shaped maze was also used for testing sensory integration between vision and audition (Fig. 4). In the visually guided task, the presentation of sample images was accompanied by sound stimuli: a circle was coupled with a meow sound and a star was coupled with a squeak sound. The presentation of sound cues was synchronized at 1 Hz with changes in the brightness of the sample images using the animation function in PowerPoint (Video 2). The sound cues were presented through a small speaker (Companion 2, Bose, Framingham, USA). In the sound cue-guided task, presentation of the sample images was omitted and the mice selected the correct branches based on sound cues only (Video 3).

Sensory integration between vision and audition was also investigated using passive exposure to sample images combined with sound cues. After the visually guided task in the absence of sound cues was completed, the mice were returned to a home cage constructed from a transparent plastic plate and placed in a dark sound-proof box. The width, length, and height of the cage were 22 cm, 32 cm, and 18 cm, respectively. Each side of the cage was surrounded by four 8-inch displays, and a speaker was set on the cage. The sample images synchronized with the corresponding sound cues were presented through the four displays and a speaker for 10 s at 30 s intervals for 48 h. The two pairs of the combined Audio-visual stimuli (“circles” plus “meow”, and “stars” plus “squeak”) were alternately presented at 30 s intervals. The memory-guided task based on Audio-visual association was tested after passive exposure.

### Two-photon imaging

Two-photon calcium imaging was performed using Cal-520 as a calcium indicator^52^. Cal-520 AM (AAT Bioquest, Sunnyvale, USA) at 8 mM was dissolved in dimethyl sulfoxide containing 20% (w/v) pluronic F-127 (Invitrogen, Carlsbad, USA), and further diluted 10 times with saline containing 0.1 mM sulforhodamine 101 (SR-101, Invitrogen). A glass pipette (tip diameter: 2–4 µm) was pulled and filled with the Cal-520 AM solution. The skull covering the right V1 was removed. The pipette was slowly inserted into the V1 and advanced into the supragranular layers, 200–250 µm deep from the pial surface. The Cal-520 AM solution was injected with a pressure of 8–25 kPa for 5–10 min, so that the cells in the area 200–300 µm from the tip of the pipette were stained. Astrocytes stained with SR-101 were excluded from further analysis. After injection, the pipette was withdrawn and the hole opened by craniotomy was covered with 2% agarose and a thin cover glass (5 × 5 mm, thickness <0.15 mm). The cover glass was firmly fixed to the skull with dental resin. Calcium imaging was performed using a two-photon microscope (TCS SP5 MP, Leica Microsystems) with a Ti-Sapphire mode-locked femto-second laser (Chameleon Vision, Coherent, Santa Clara, USA). Images were obtained via a ×20 water immersion objective lens (numerical aperture 1.0, HCX PL APO, Leica Microsystems). The excitation wavelength for Cal-520 was 900 nm, while that for SR-101 was 940 nm. Observations were started 40–50 min after Cal-520 AM injection. Cal-520 fluorescence was observed between 500 and 550 nm. Circular windows with a diameter of 20 pixels (14.3 μm) were placed on neuronal cell bodies stained with Cal-520 but not with SR-101, and the obtained fluorescence signals in the windows were averaged across 10 trials. The normalized fluorescence changes in ΔF/F0 were calculated using the averaged baseline intensity (F0) collected immediately before stimulus onset.

### Statistical analysis

Paired data obtained from the same mice were evaluated using the Wilcoxon signed rank test. Unpaired data obtained from different mice were evaluated using the Mann Whitney U-test. Values in the figures represent the mean and SEM in groups of mice, unless otherwise specified. The cumulative distribution of the orientation selectivity index or direction selectivity index in different neurons was evaluated using the Kolmogorov-Smirnov test. Deviation of behavioral performance from chance level (50%) was evaluated with the χ^2^-test in five successive sessions (100 trials).

## Electronic supplementary material

Supplementary Figures

Video 1

Video 2

Video 3
