# Supplementary material for: Molecular diversity of clustered protocadherin-α required for sensory integration and short-term memory in mice
Source: Sci Rep. 2018 Jun 25;8:9616. doi: 10.1038/s41598-018-28034-4 (PMC6018629; doi:10.1038/s41598-018-28034-4)
Supplement: Supplementary file 1 — Supplementary Figures [file 41598_2018_28034_MOESM1_ESM.pdf]

SUPPLEMENTARY FIGURES

Molecular diversity of clustered protocadherin-α required for sensory integration and short-term memory in mice

Tatsuya Yamagishi[1,2*], Kohei Yoshitake[1,4*], Daiki Kamatani[1,4], Kenji Watanabe[1,4], Hiroaki Tsukano[1], Ryuichi Hishida[1], Kuniyuki Takahashi[2], Sugata Takahashi[2], Arata Horii[2], Takeshi Yagi[3,4], Katsuei Shibuki[1,4]

*These two researchers contributed equally to the study.

1. Department of Neurophysiology, Brain Research Institute, Niigata University
   1-757 Asahi-machi, Chuo-ku, Niigata 951-8585, Japan
2. Department of Otolaryngology Head and Neck Surgery, Graduate School of Medical and Dental Sciences, Niigata University
   1-757 Asahi-machi, Chuo-ku, Niigata 951-8510, Japan
3. KOKORO-Biology Group, Graduate School of Frontier Biosciences, Osaka University
   1-3 Yamadaoka, Suita 565-0871, Osaka, Japan
4. The Japan Agency for Medical Research and Development
   1-3 Yamadaoka, Suita 565-0871, Osaka, Japan

Correspondence: Katsuei Shibuki, Department of Neurophysiology, Brain Research Institute, Niigata University, 1-757 Asahi-machi, Chuo-ku, Niigata 951-8585, Japan

Phone: +81-25-227-0625; Fax: +81-25-227-0628; E-mail: shibuki@bri.niigata-u.ac.jp

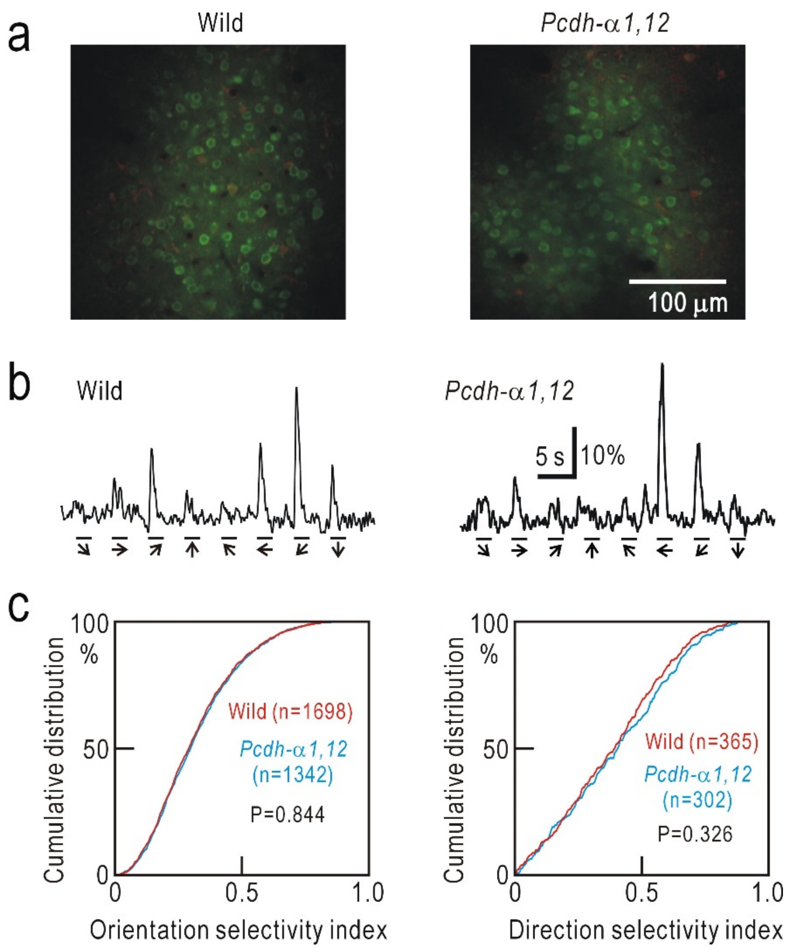

Supplementary Figure S1. Selectivity of V1 neurons.

a, Neurons stained with Cal-520 but not with SR-101 in the V1 of a wild-type mouse (left) and a *Pcdh-α1,12* mouse (right). The image was obtained using a two-photon microscope. **b,** Sample traces of neuronal calcium responses to moving grating patterns in eight directions (from -45° to 270° in 45° steps) for 2 s in a wild-type mouse (left) and a *Pcdh-α1,12* mouse (right). **c,** Cumulative distributions of the orientation selectivity index (OSI, left) and direction selectivity index (DSI, right) of neurons obtained from three wild-type mice and three *Pcdh-α1,12* mice. The OSI was obtained from1698 and 1342 neurons, respectively. The DSI was obtained from 365 and 302 neurons with an OSI > 0.45, respectively. There was no significant difference in the cumulative distribution of the OSI or DSI between wild-type and *Pcdh-α1,12* mice.

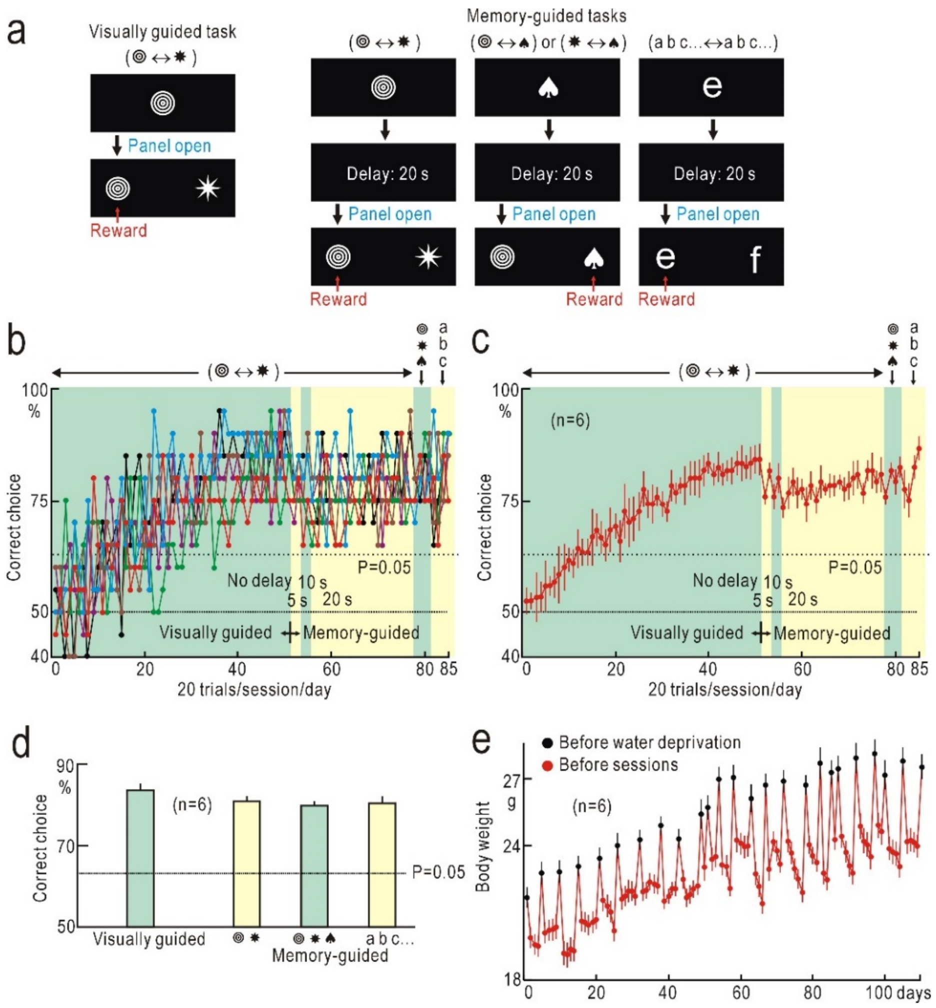

Supplementary Figure S2. Visual discrimination ability of mice.

a, Tasks in this experiment. Matching-to-sample tests with a delay of 0–20 s were used. After discrimination between circles and stars was completed, discrimination between circles and spades or stars and spades, and between alphabet pairs was tested. **b,** Performance in wild-type mice. Dotted line at approximately 63% performance shows a significant (P = 0.05) deviation of the averaged performance from chance level in four successive sessions. **c,** The mean and SEM of data shown in (**b**). **d,** Averaged performance in the visually guided task and memory-guided tasks with a delay of 20 s discriminating between circles and stars, between circles and spades or stars and spades, and between alphabets. **e,** Body weight change during the experiments. Black dots and bars represent data before water deprivation, and red dots and bars show data before each session.

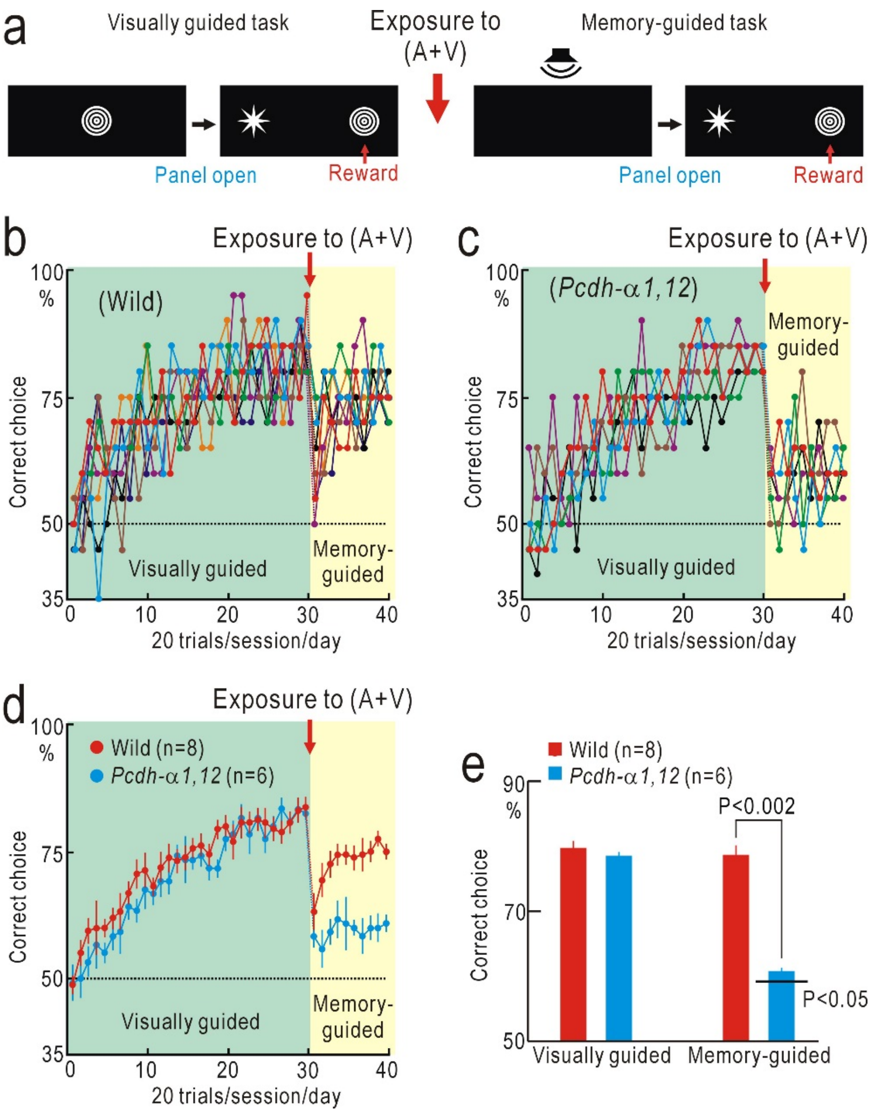

Supplementary Figure S3. Audio-visual associative learning after passive exposure to audio-visual stimulation.

a, Visually guided and memory-guided tasks. Mice were exposed to audio-visual stimulation for 48 h between the two tasks. **b,** Performance of eight wild-type mice. In this experiment, sound cues were combined with visual stimuli only during passive exposure for 48 h between the two tasks. **c,** Performance of six *Pcdh-α1,12* mice. **d,** The mean and SEM. of data shown in (**b**) and (**c**). **e,** Averaged amplitudes of performance in the last five sessions of the visually guided and memory-guided tasks. The performance of *Pcdh-α1,12* mice in the memory-guided task was significantly worse than that of wild-type mice.

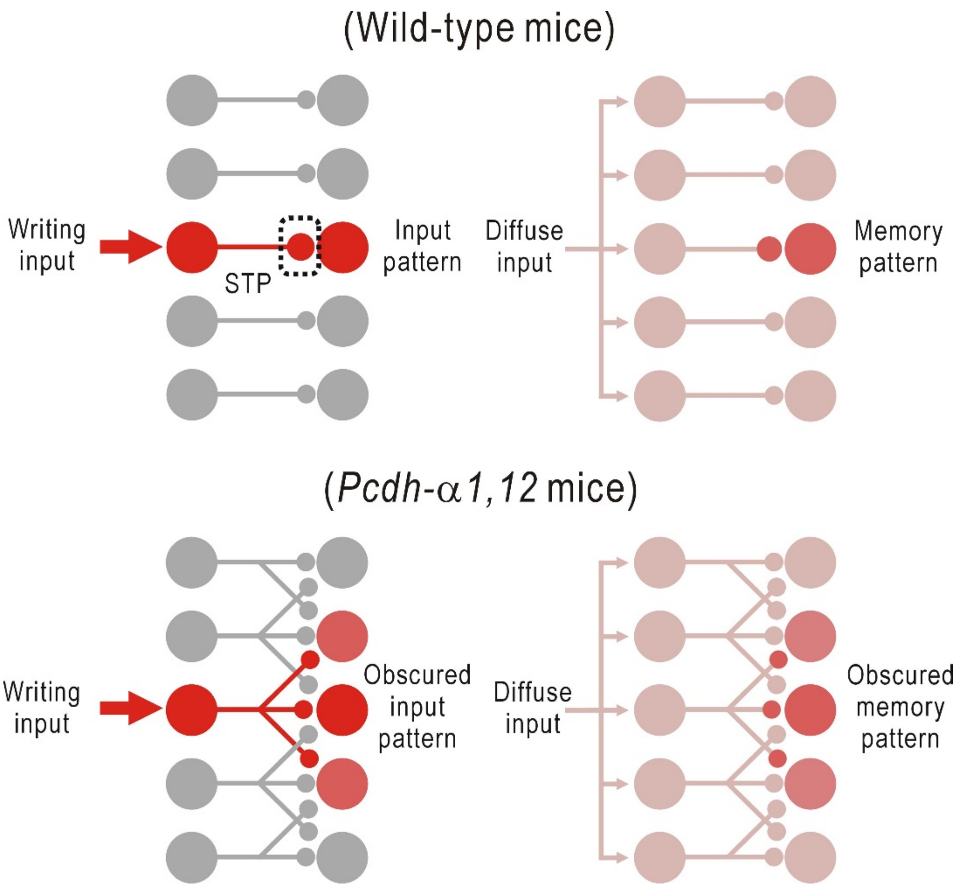

Supplementary Figure S4. Sparse circuit model of short-term memory.

In wild-type mice, localized writing input to the first layer produces short-term potentiation (STP) in the stimulated synapses. The information stored as STP can be converted to short-term memory by adding diffuse input to the first layer. In *Pcdh-α1,12* mice, the sparseness of the circuits is slightly deteriorated, thus the content in short-term memory is obscured.
